# IgG4-related Disease: a diagnostic challenge

**DOI:** 10.4322/acr.2021.312

**Published:** 2021-08-20

**Authors:** Rodrigo Díaz Olmos, Marcelo Arlindo Vasconcelos Miranda Rodrigues, Cristiane Rúbia Ferreira, Rita de Cássia Franco Etrusco, Carla Romagnolli

**Affiliations:** 1 Universidade de São Paulo (USP), Hospital Universitário, Internal Medicine Division, São Paulo, SP, Brasil; 2 Universidade de São Paulo (USP), Hospital Universitário, Faculty of Medicine, Internal Medicine Department, São Paulo, SP, Brasil; 3 Universidade São Caetano do Sul (USCS), Faculty of Medicine, São Paulo, SP, Brasil; 4 Universidade de São Paulo (USP), Hospital Universitário, Anatomic Pathology Service, São Paulo, SP, Brasil; 5 Universidade de São Paulo (USP), Hospital das Clínicas, Faculty of Medicine, São Paulo, SP, Brasil

**Keywords:** Immunoglobulin G, Immunoglobulin G4-Related Disease, Lymph Node Excision

## Abstract

Immunoglobulin IgG4-related disease (IgG4-RD) is an immune-mediated fibroinflammatory condition with a characteristic histopathological appearance that can affect almost any organ. The clinical features result from a focal or diffuse appearance of a tumor-like swelling of the affected organs, identified by physical and/or imaging examination. Herein, we report the case of a 38-year-old male complaining of a worsening chronic right lumbar pain associated with legs and scrotum edema. He also had itchy and erythematous cutaneous lesions on the abdominal wall over the last 8 months, and complained of a diffuse and mild to moderate abdominal discomfort. On examination, the liver was firmly enlarged and tender. His legs had 2+ symmetrical pitting edema extending from his feet to just above the knees. An abdominal computed tomography scan showed a large mass (10 x 8 x 4cm) involving the abdominal infrarenal aorta and the iliac arteries, and compressing the inferior vena cava, with dilated iliac veins, raising the possibility of lymphoproliferative disease. During the initial investigation, the laboratory workup revealed anemia, without other marked changes. A laparoscopic-guided biopsy of the peri-aortic mass was undertaken. The histological report associated with IgG4 immunoglobulin measurement rendered the diagnosis of IgG4-RD. The patient had a favorable outcome after the use of glucocorticoids with the abdominal mass remission.

## INTRODUCTION

Immunoglobulin IgG4-related disease (IgG4-RD) is an immune-mediated fibroinflammatory condition with a characteristic histopathological appearance that may involve virtually all organs.**^1^** It was initially identified in the pancreas in 2001 (type 1-autoimmune pancreatitis or pancreatitis related to IgG4), but it was not recognized as a systemic condition until 2003, when extrapancreatic manifestations were identified in patients with type 1-autoimmune pancreatitis**.** **^1^** The most frequently affected organs include the major salivary and lacrimal glands (sclerosing sialadenitis), orbital disease, autoimmune pancreatitis, retroperitoneal fibrosis (commonly with chronic peri-aortitis and the ureters involvement), biliary tract (sclerosing cholangitis), lymph nodes and kidneys (tubulointerstitial nephritis).**^2^** **^,^** **^3^** The IgG4-related dacryoadenitis and sialadenitis may be more common in women; atopic dermatitis, asthma, or chronic sinusitis may be present in up to 40% of patients; multiple and simultaneous organ involvement occurs in 50% of cases; and it most commonly affects middle-aged to elderly individuals, with a male to female ratio of 4:1. These multiple organ involvements share several core pathologic features, as well as clinical and serologic similarities. In this setting, IgG4-RD could be compared with sarcoidosis because of the multiorgan involvement with a unique histological appearance.

The pathologic features include tumor-like swelling of the involved organs, a lymphoplasmacytic infiltrate with IgG4-positive plasma cells, obliterative phlebitis, and a variable degree of fibrosis that has a characteristic "storiform" pattern. Furthermore, there is often but not always, elevated serum IgG4 determination. Table 1 shows the clinical, laboratory, and pathological criteria for the diagnosis of IgG4-RD.^2^

**Table 1:** Clinical, laboratory, and pathological criteria for IgG4-RD

•swelling or mass in one or more organs;
•serum IgG4 greater than 1.35 g/L;
•histopathological features:
◦ marked infiltration by lymphocytes and plasma cells,
◦ more than ten IgG4-positive plasma cells per high-power field with an IgG4/IgG ratio greater than 40%,
◦ storiform fibrosis.
◦obliterating phlebitis

In the past few decades, this entity has been recognized as a systemic disease encompassing many individual organ diseases that were not known to be related (and are now part of the spectrum of IgG4-related disease), such as retroperitoneal fibrosis, Mikulicz’s syndrome, Küttner’s tumor, and Riedel thyroiditis.

The pathogenesis of IgG4-RD is unclear; however, it seems to be involved in an allergic mechanism. The T-helper 2 (Th2) cytokine expression and regulatory cytokines have been noted to be up-regulated in the affected tissues of patients with IgG4-RD. The increase in Th2 cytokines (IL-4, -5, -10, and -13, and transforming growth factor-b) might have a role in the progression of fibrosis, which is characteristic of this disease.

The diagnosis of IgG4-RD might be easily missed by unsuspecting general practitioners, internists, hematologists, and rheumatologists, as patients may present with a multitude of clinical presentations that may mimic some hematologic, infectious, and rheumatologic diseases such as Castleman disease, lymphoma, plasma cell neoplasms, hypereosinophilic syndromes, sicca syndrome, systemic lupus erythematosus, infectious mononucleosis and so forth. Therefore, it is crucial for the clinicians who first see these patients to suspect this entity when facing cases with lymphadenopathy, eosinophilia, and polyclonal hypergammaglobulinemia together with some of the more common presentations such as lacrimal and salivary glands involvement, orbital disease, and tumor-like swelling of the involved organs.**^4^** The clinical manifestations generally result from a focal or diffuse appearance of tumor-like swelling of the organs, identified by physical and/or imaging examination. The clinical course is normally subacute, and constitutional symptoms are uncommon.

## CASE REPORT

A 38-year-old male presented to the emergency department with worsening chronic right lumbar pain associated with legs and scrotum edema. He also had itchy and erythematous cutaneous lesions on the abdominal wall over the last 8 months, with no remission despite numerous therapeutic attempts, and complained of diffuse mild to moderate abdominal pain. He denied weight loss, fever, or night sweats. He was a smoker, and his past medical history included unilateral (right) polycystic kidney disease with functional renal exclusion and a left vicariant kidney (he was waiting for a right nephrectomy) and pulmonary tuberculosis in 2003. He frequently used diclofenac, paracetamol, caffeine, and carisoprodol for his chronic lumbar pain.

On admission, he had a good general appearance, no signs of respiratory distress, no alterations in skin color. His heart rate was 95 bpm, blood pressure 166 x 104 mmHg, and room air oximetry were 96%. There were eczematous desquamating skin lesions on his thighs and legs. There were no enlarged lymph nodes. Chest auscultation revealed decreased breath sounds and dullness to percussion at the right lung base. On abdominal palpation, the liver was enlarged, palpable up to 5 cm below the costal margin and was slightly tender. His legs had 2+ symmetrical pitting edema extending from his feet to just above his knees. Laboratory workup revealed mild anemia (Hb 10.8 g/dl), normal white blood cell, and platelet count. The hepatic enzymes, bilirubin, creatine kinase, lactate dehydrogenase, uric acid, and electrolytes were within the normal range. However, the creatinine was 1.47 g/dL (RR: 0.70 - 1.20 g/dL) and the creatinine clearance estimated by the CKD-EPI equation was 69 mL/min/1.73m^2^. C-reactive protein (32 mg/L; RR: < 3 mg/L) and erythrocyte sedimentation rate (46 mm/1h) were increased. HIV, HCV, syphilis, and Hepatitis B virus serologies yielded negative results. The rheumatoid factor and antinuclear antibodies were negative, and the complement was normal. Urinalysis revealed 1+ of protein and 150 x 10^3^ red blood cells/mm^3^.

**Figure 1:**
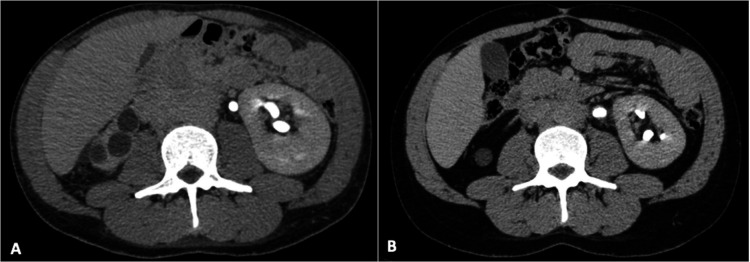
Abdominal CT axial plane: **A** – Initial examination on admission. Note large mass involving the abdominal aorta; **B** – CT scan performed 7 months after the first examination and under treatment with corticosteroid. Note the expressive reduction of the periaortic mass.

Abdominal computed tomography (CT) showed a large, contrast-enhanced mass (10 x 8 x 4cm) involving the abdominal infrarenal aorta and the iliac arteries and compressing the inferior vena cava with dilated iliac veins and the left ureter (Figure 1A), raising the hypothesis of the lymphoproliferative disease. The liver had normal size but had a heterogeneous attenuation.

The chest CT showed a diffuse pleural thickening in the right hemithorax with a small loculated pleural effusion, and enlarged mediastinal and peri-esophageal lymph nodes.

**Figure 2:**
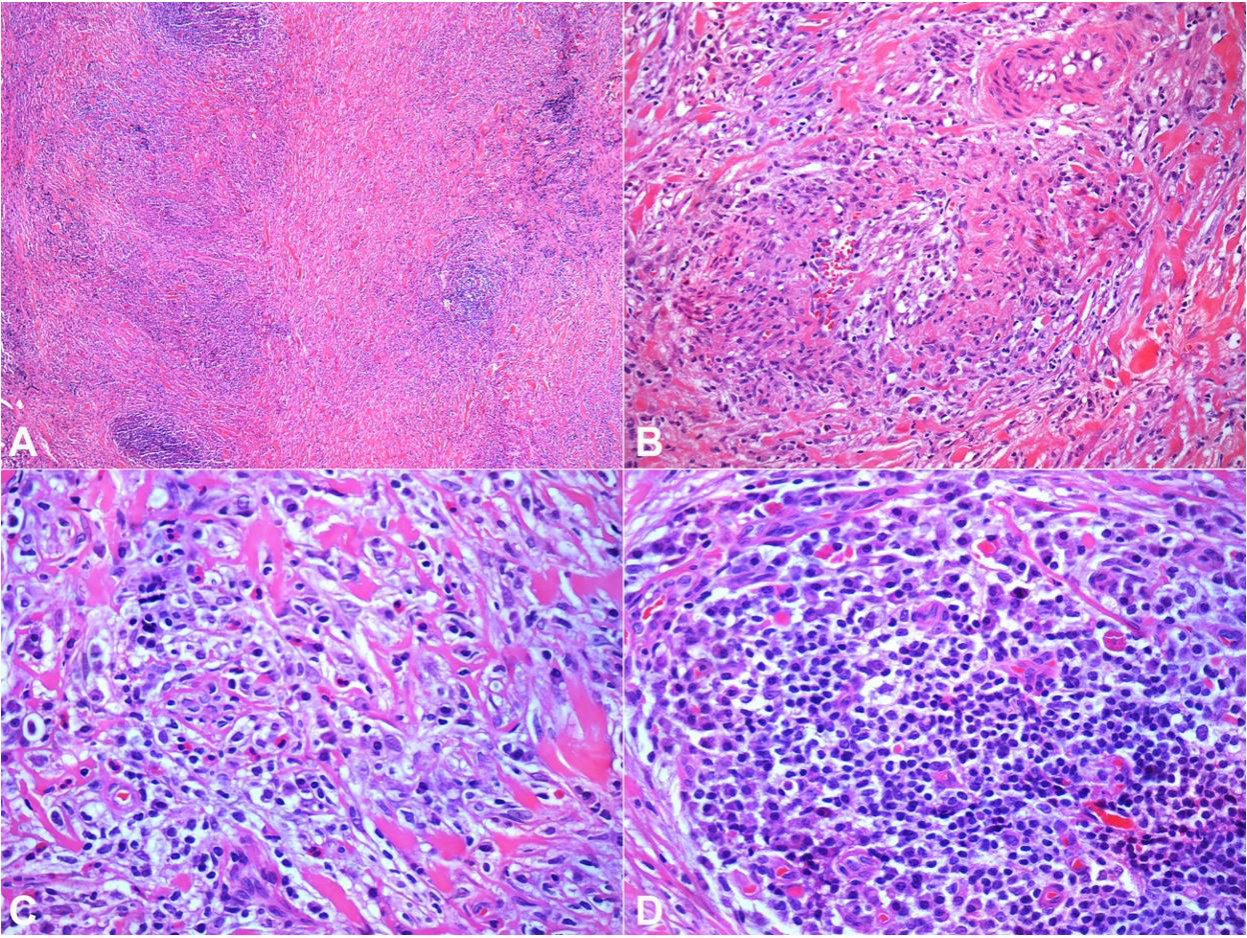
Photomicrographs of the biopsy: **A** – Fibro conjunctive tissue exhibiting fibrosis, with thick collagen bundles and moderate inflammatory infiltrate with lymphoid aggregates (HE – 40X); **B** – Obliterating phlebitis (HE – 200X); **C** – Fibro conjunctive stroma showing thick collagen bundles with few eosinophils, histiocytes and small lymphocytes mixed with plasma cells (HE – 400X); **D** – Numerous plasma cells surrounding a lymphoid aggregate (HE – 400X).

**Figure 3:**
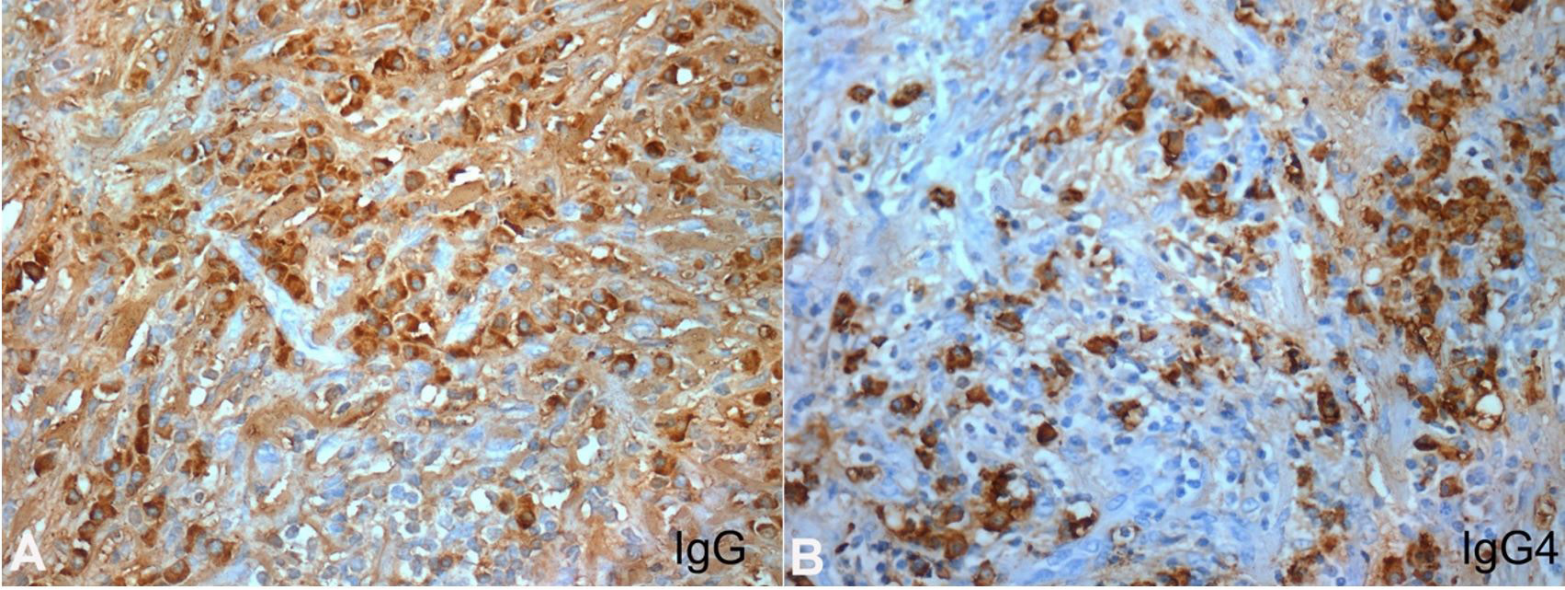
Photomicrographs of the Immunohistochemical staining of the biopsy: **A** – Numerous IgG positive plasma cells near a lymphoid aggregate (400X); **B** – Increased number of IgG4 positive plasma cells presenting an elevated IgG4/IgG ratio greater than 30% in areas of more density (400X).

The patient was admitted with the working diagnosis of a lymphoproliferative disease for a confirmatory diagnostic workup. A double J ureteral stent was placed in the left ureter. A CT-guided biopsy of the right pleural thickening was performed, which lacked a precise diagnosis. The patient was submitted to a laparoscopic-guided biopsy of the peri-aortic mass. This biopsy showed a fibro-connective tissue exhibiting fibrosis, obliterating phlebitis, and moderate mixed inflammatory infiltrates with few eosinophils, histocytes, and small lymphocytes and frequent plasma cells (Figures 2 and 3).

The immunohistochemical examination (Table 2) showed an increased number of IgG4 positive plasma cells. The IgG4/IgG ratio of 30% and approximately 80 IgG4 positive-plasma cells per high-power field (HPF) in areas of more density. This examination, together with the morphological and clinical findings, is highly suggestive of IgG4-RD.

**Table 2:** Immunohistochemical examination

**Antibodies**	**Result**	**Antibodies**	**Result**
AE1/AE3	Negative	CD68	Positive (in histocytes)
S-100	Inconclusive	Lysozyme	Positive (focal in histocytes)
SMA	Positive	CD1a	Negative
Desmin	Negative	CD23	Positive (focal in follicular dendritic cells)
CD117	Negative	CD43	Positive (reactive T-lymphocytes)
CD20	Positive (reactive B-lymphocytes)	Kappa	Inconclusive
CD3	Positive (reactive T-lymphocytes)	Lambda	Inconclusive
CD30	Negative	IgG	Positive (frequent plasma cells
AlK c	Negative	IgG4	Positive (frequent plasma cells

The patient was started on 1.0 mg/kg/day of prednisone for 2 weeks and then tapered for 0.6 mg/kg/day for two months, and to 20 mg/day for 3 months, afterward. The outcome was favorable and he gradually recovered his previous health status. The abdominal and lumbar pain ceased in about 3 weeks after starting treatment, his hemoglobin level returned to normal and his inflammatory markers decreased. Five months after initiation of corticotherapy, a new abdominal CT scan was done (Figure 1B), and the initial contrast-enhanced mass had almost disappeared, but it showed an inferior vena cava thrombosis extending to bilateral external iliac veins. He was started on warfarin. The double J ureteral stent was removed, and the patient remains asymptomatic with a good urine output with no renal function impairment. At the closure of this manuscript, he was asymptomatic and taking only 5 mg/day of prednisone.

## DISCUSSION

Retroperitoneal fibrosis/periaortitis, as in this case, affects approximately 9.6–27.0% of the cases of IgG4-RD 5 ^-^ 12. It usually manifests with abdominal, flank, or lumbar pain, edema of the lower limbs, decreased urinary output, low-grade fever, loss of appetite, and weight loss. Hydronephrosis occurs in 33–67%, being mostly (75%) unilateral, similar to the involvement of former idiopathic retroperitoneal fibrosis^13^^,^ 14 ^.^

The diagnosis of IgG4-RD is challenging. No single marker or clinical feature is specific to make a definitive diagnosis of the disease. Thus, the diagnosis is made based on clinical, laboratory, histological and radiological criteria. The involvement of many organs and the wide range of clinical manifestations, depending on the affected organ, make the diagnosis even more difficult. Although the serum IgG4 concentration is generally high (and even higher in atopic patients) in patients with IgG4-RD, in up to 40% of the biopsy-confirmed cases the IgG4 serum level is normal. Thus, the serum IgG4 determination within the normal range does not exclude the diagnosis^9^^,^ 15 ^,^ 16 ^.^ Similarly, a high serum level of IgG4 is not enough to confirm the diagnosis, because several infectious, neoplastic, and inflammatory conditions can also lead to similar results.^1^^,^ 2 ^,^ 4

The three main histopathological characteristics associated with IgG4-RD are (i) dense lymphoplasmacytic infiltrate; (ii) storiform fibrosis; and (iii) obliterating phlebitis. A reliable pathological diagnosis of IgG4-RD requires the presence of at least two of these criteria, which in most cases are the dense lymphoplasmacytic infiltrate associated with the storiform fibrosis. 2 The lymphoplasmacytic infiltrate is rich in IgG4+ plasma cells. Other non-specific features associated with IgG4-RD are phlebitis without obliteration of the lumen and an increased number of eosinophils.

Some studies^17^^–^ 19 recently proposed the diagnostic criteria for IgG4-RD: (i) evidence of nodules or masses, local or diffusely spread in one or multiple organs; (ii) elevation of serum IgG4 (> 135mg / dL); and (iii) tissue infiltration of plasma cells IgG4 +> 10 /high power field and IgG4 + / IgG + cells> 40%. The presence of the three criteria indicates a “definitive diagnosis of IgG4-RD”. If criteria 1 and 3 are met, even in the absence of other histopathological findings suggestive of the disease, it is defined as “possible diagnosis of IgG4-RD” and, in patients with organic involvement and with histopathological criteria present, but without increased serum IgG4 concentration, the diagnosis is considered as “probable diagnosis of IgG4-RD”.

The treatment of IgG4-RD depends on the affected organ, ranging from expectant management (lymphadenopathies and asymptomatic pulmonary nodules) to aggressive treatment with glucocorticoids in case of vital organs at risk of dysfunction**.** **^1^** Glucocorticoid is the first-line drugs to induce remission. The recommended dose and time of treatment vary in the literature. The treatment result is observed quickly. Symptoms improve in one month, and the serum IgG4 concentrations decrease rapidly, permitting the glucocorticoid withdrawal within a few weeks, in most patients.^22^^–^ 24 The initial dose and duration of glucocorticoid therapy should be carefully evaluated based on the comorbidities of each patient and their potential for medication intolerance.^22^^–^ 24 The conventional steroid-sparing agents are ineffective for the treatment of IgG4-RD, and no benefit in relapse-free survival has been observed by adding these medications together with the glucocorticoids.^24^ As they are ineffective, they cannot be used in other situations, such as corticosteroid contraindication or refractoriness. Azathioprine, methotrexate, or mycophenolate mofetil can be used in case of glucocorticoid usage contraindications^21^^–^ 24. Rituximab is also the option in recurrent or refractory cases.^21^^,^ 24 Early diagnosis and treatment prevent organ dysfunction by fibrosis and poor therapeutic response.^20^^,^ 21 Thus, the degree of fibrosis at the beginning of treatment is still the main predictor of the therapeutic success.^20^^,^ 21 ^,^ 24 During the follow-up, after starting treatment, especially with glucocorticoids, it is expected a decrease in the IgG4 levels; however, there may be clinical remission even without its fall, as well as recurrence with normal IgG4 levels.^1^

## CONCLUSION

IgG4-RD can be a challenging diagnosis and be a differential diagnosis of intra-abdominal mass. In the case presented herein, it was necessary to perform a biopsy of the intra-abdominal mass and IgG4 measurement. It is important to consider this differential diagnosis in similar cases. In our case, the response to corticotherapy was favorable, as shown in the literature.
